# Prospective evaluation of clinical and radiographic 10-year results of Fitmore short-stem total hip arthroplasty

**DOI:** 10.1186/s13018-023-04359-3

**Published:** 2023-11-23

**Authors:** Jana F. Schader, Caroline Thalmann, Katharina S. Maier, Tom Schiener, Karl Stoffel, Arno Frigg

**Affiliations:** 1 Department of Orthopaedic Surgery, Cantonal Hospital Graubuenden, 7000 Chur, Switzerland; 2 University of Basel, 4001 Basel, Switzerland; 3 Department of Orthopaedic Surgery, University Hospital Basel, 4031 Basel, Switzerland

**Keywords:** Total hip arthroplasty, Fitmore, Clinical results, Radiographic results, Long-term, Short stem

## Abstract

**Background:**

Short stems were introduced into total hip arthroplasty (THA) to preserve bone stock, to transmit more load to the proximal femur, and to enable minimal invasive approaches. This study is the first long-term study (with a follow-up of 10 years) of the survival as well as the clinical and radiographic outcomes of the Fitmore hip stem, a short curved uncemented stem.

**Methods:**

In total, 123 Fitmore hip stems were prospectively evaluated. At the final 10-year follow-up, 80 Fitmore stems (78 patients: 30 female, 48 male) were eligible for evaluation. Clinical parameters were thigh pain, EQ-5D, Harris Hip Score (HHS) and Oxford Hip Score. Radiographic parameters were cortical hypertrophy (CH), bone condensation, cortical thinning, radiolucency, reactive lines, calcar rounding, calcar resorption, subsidence and varus/valgus position.

**Results:**

After 10 years, there was a survival rate of 99% (1 revision because of aseptic stem loosening). HHS had improved from 59 to 94 and Oxford Hip Score from 22 to 43. CH rate after 1 year was 69% and after 10 years 74%. In the first year, radiolucency was found in 58% and in 17.5% after 10 years. Subsidence after 1 year was 1.6 ± 1.6 mm and 5.0 ± 3.1 mm after 10 years.

**Conclusions:**

The Fitmore hip stem showed a survival rate of 99% as well as good clinical and radiographic outcomes in the long-term follow-up of 10 years.

## Background

Total joint arthroplasty has been hailed as the surgical technique of the century, with excellent overall long-term outcomes when using established implants [1]. Part of the success might be due to constant development of these implants resulting in improvement of structural strength, decrease in wear, and many different design concepts like long and short stems as well as resurfacing. Short stems appear to provide larger bone stock preservation for potential revision surgery and improve load transfer to the proximal femur [2–4]. Finite-element studies showed short-stem implants significantly reduced stress shielding in the calcar region compared to conventional long-stem implants [5, 6]. Furthermore, short-stem implants enable less invasive surgical interventions and thereby lower the risk of intraoperative periprosthetic fractures, hematomas, and wound healing problems [7].

**Fig. 1:**
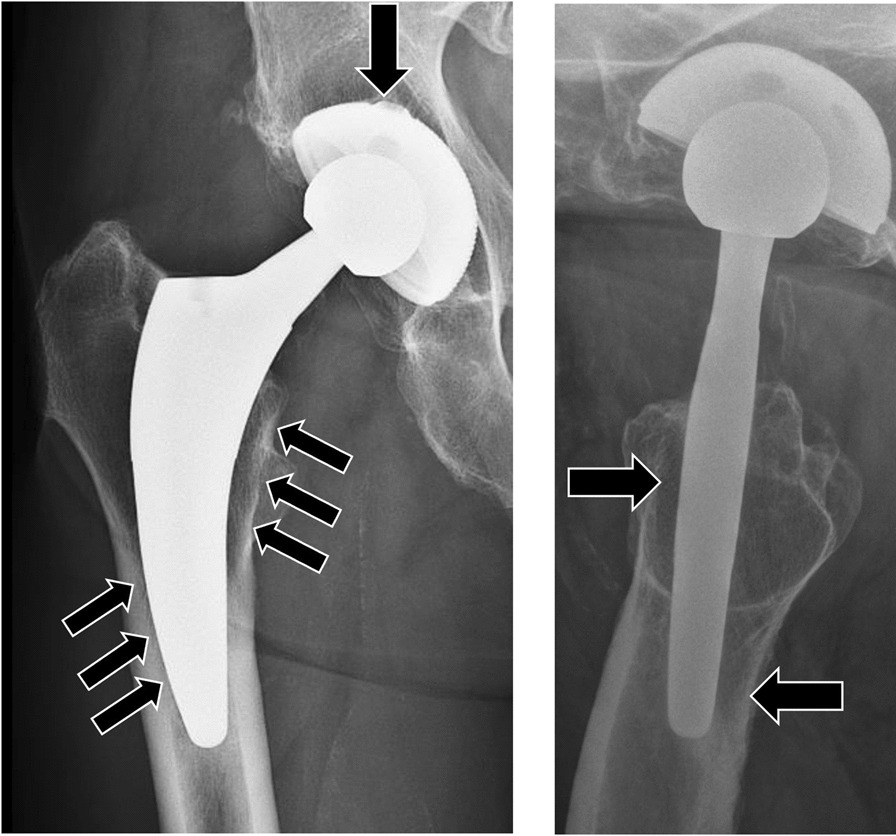
Radiographs anteroposterior (ap) and axial of the Fitmore hip stem position in the femoral shaft. Load transfer contact points at the implant-bone interface indicated via arrows at the femoral head element; calcar; and anterior/posterior subtrochanteric shaft

The Fitmore hip stem (Zimmer Biomet, Winterthur, Switzerland) uses the concept of a short, uncemented curved stem (Fig. 1). Primary stability is achieved by a press-fit fixation and a triple-tapered design that results in an even load distribution [2, 4]. The shortened and flattened stem aims to preserve cancellous bone by following the anatomical pathway. Furthermore, this stem is thought to gain rotational stability from contact in the calcar region. However, by acting more rigidly than longer stems, it may induce remodeling of the periprosthetic bone structure resulting in radiographic alterations like cortical hypertrophy [2]. Short- and midterm clinical outcomes showed excellent results with an improvement of Harris hip score (HHS) from preoperatively 60 to 99 after 2 years [8] and from 59 to 94 after 5 years [9]. Survival rates of 100% after 2 years [10] and 99% at 5 years [9] were reported.

Currently, there is no study published with a minimal 10-year follow-up of the Fitmore stem. Therefore, the primary aim of this study was to obtain survival, clinical and radiographic outcomes of the Fitmore hip stem in a long-term follow-up of 10 years.

## Materials and methods

This prospective study was approved by the local Ethical Review Board and all patients have provided written informed consent. The study was carried out in accordance with the Declaration of Helsinki and the applicable laws. From April 2008 to 2010 a total of 123 primary THA were performed in 120 patients using the Fitmore hip stem (Zimmer Biomet, Winterthur, Switzerland, Fig. 1).

Study participants were included if they had received the Fitmore hip stem in the time frame of the study, and if complete clinical and radiographic data preoperatively and for all postoperative follow-up visits (after 1, 2, 3, 5 and 10 years) were available. Exclusion criteria were: lost to follow-up (n = 14), death (n = 10), missing data (n = 5) and refusal to participate (n = 12). In total, 81 Fitmore hip stems were included in the study at baseline for the survival rate. At the final 10-year follow-up, 80 Fitmore hip stems (78 patients: 30 female, 48 male) were eligible for evaluation (Table 1); 1 stem was excluded from the 10-year results because it underwent a stem related revision (conversion to a CLS stem due to aseptic loosening). Patient age was 61 ± 10 years (mean ± standard deviation (SD)). Indications for THA in the initial study collective were primary osteoarthritis (92%), fracture (3%), osteonecrosis (2%), post-traumatic arthritis (2%), and hip dysplasia (1%).

**Table 1:** Patient-, implant- and surgical data

Demographic data	
N	80 hips (78 patients)
Mean age at time of operation (years)	60.7 (± 10.2)
Gender (M/F)	61.3% / 38.8%
BMI/Body mass index (kg/m^2^)	27.3 ± 4.8
Dorr Index (A/B/C)	82.5%/17.5%/0%
Fitmore hip stem family (A/B/B extended/C)	8.8%/52.5%/36.2%/2.5%
CCD angle (°)	134.1 (± 4.3)
Median stem size (median, range)	7 (5–8)
Mean preoperative leg length difference	− 3.0 (± 5.3)
Mean postoperative leg length difference	− 1.0 (± 6.4)
Mean difference between preoperative and postoperative leg length difference	1.8 (± 5.2)
Mean preoperative offset	38.5 (± 7.9)
Mean postoperative offset	40.5 (± 7.6)
Mean difference between preoperative and postoperative offset	2.0 (± 7.6)
Varus/neutral/valgus position after surgery	0%/82.5%/17.5%

Values are mean (± SD)

Surgeries were performed in a large general hospital. In all cases with no contraindications except decreased bone quality (Dorr type C) on the preoperative radiographs, the Fitmore stem was used as primary standard implant. All surgeries were performed by a small team of orthopedic surgeons. Therefore, surgical techniques, pre- and postoperative management, as well as anesthesiologic standards were quite consistent.

In 80%, an anterolateral, minimally invasive approach was used and in 20% of cases a direct lateral approach (Hardinge, transgluteal) according to the surgeon’s preference. In 90% a Fitmore cup was used. In 10% of cases the bone quality was too low for a press-fit of the Fitmore cup and a trabecular metal modular cup was used with a CoCr head (Zimmer Biomet, Winterthur, Switzerland). The following acetabular liners were used: Alpha Durasul, Standard 110 (89%), Alpha Sulene PE 1 (1%), Trilogy Longevity, XLPE, 3.5 mm Offset 2 (2%), Trilogy Longevity, XLPE, 3.5 mm Offset, 10 Degree Elevated Rim 10 (8%). All patients started full weight-bearing activities with a 4-point crutch gait immediately after surgery.

### Prospective clinical evaluation

Patient data were clinically documented at baseline, immediately postoperatively, and followed-up after 1, 2, 3, 5 and 10 years in a physical examination, documenting thigh pain, EQ-5D, HHS, and Oxford Hip Score. At each visit, antero-posterior (AP) and axial radiographs were taken with internally rotated legs. Radiographs were standardized by placing a 25-mm radiopaque gage ball between the thighs of the patient (at baseline) and by using the implant head diameter according to type size (for postoperative radiographs).

### Radiographic evaluation

**Fig. 2:**
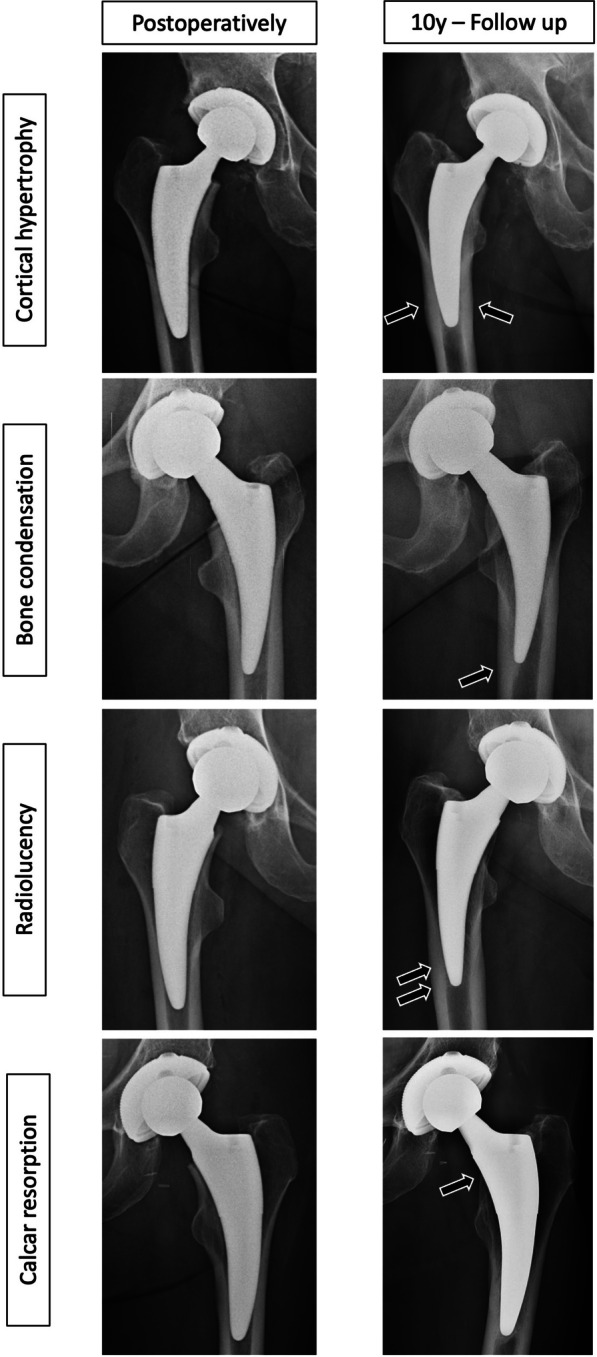
Radiological outcome parameters on ap radiographs immediately postoperatively and after 10 years: Radiolucent lines, calcar resorption, bone condensation and cortical hypertrophy

Radiographic measurements of 10-year follow-up were performed using a PACS-Web-Viewer program (GE Healthcare, Solingen, Germany). Two different investigators assessed all radiographs, measures on which both disagreed were verified by another independent surgeon. The following parameters were measured in the 10-year follow-up (Fig. 2): (1) cortical hypertrophy (CH), i.e., distance between the inner to the outer edge of the cortical bone perpendicular to the stem axis; (2) bone condensation, i.e., the reaction of cancellous bone to stem implantation which is visible as a radiographically denser area, usually located below the tip of the stem; (3) cortical thinning; (4) radiolucency; (5) reactive lines; (6) calcar rounding; (7) calcar resorption; (8) subsidence, i.e., the difference between the level of the shoulder of the implant and a parallel line of the tip of the greater trochanter in AP radiograph (follow-up radiographs were referenced to the immediate postoperative ones); and (9) varus/valgus position, i.e., the angle between the axis of the stem defined as the most distal point of the stem and the midway point between stem shoulder and outer stem neck and the femur. The neutral position was defined as 0° ± 5°, higher positive values as varus, and higher negative values as valgus.

### Statistics

Standard statistical methods were used for general data analysis using R programming language (version 3.3.3) [11]. The package *Tableone* was applied for description of baseline and clinical characteristics. Patient parameters (age, gender, Dorr Index), implant parameters (stem type, stem size), surgical parameters (leg length difference, offset, varus/valgus), radiographic parameters, and clinical outcome parameters were analyzed descriptively. Mean and standard deviation are reported. Patient, implant, and surgical parameters (Table 1) were analyzed for correlation with radiographic parameters (Table 3) and clinical outcome parameters (Table 2). Radiographic parameters (Table 3) were analyzed for their effect on clinical outcome. Binary outcome parameters assessed in AP and axial radiographs were collapsed into one single variable: “yes” in either AP or axial was coded as “yes,” and “no” in both AP and axial was recoded as “no.” Categorical variables were compared with a chi-square test, and continuous variables were compared with a Wilcoxon rank sum test (for two groups) or a Kruskal–Wallis test (for more than two groups). Kendall’s rank correlation was computed for two ordinal variables. No correction for multiple tests was made in this explorative study. The level of statistical significance was set to 0.05 for all tests.

**Table 2:** Clinical parameters after 10 years follow-up

Clinical parameter	
Thigh pain (none/slight/moderate/severe)	85.9%/12.7%/1.4%/0%
EQ5D-severity of pain (none/moderate/excessive)	69.7%/27.3%/3.0%
Mean EQ5D-score^a^	0.9 ± 0.2
Health state	81.1 ± 15.7
Mean Harris hip score^a^	94.3 ± 7.3
Oxford hip score	43.0 ± 5.5
Oxford-severity of pain (none/very mild/mild/moderate/severe)	51.5%/20.6%/22.1%/4.4%/1.5%

^a^Values are mean (± SD)

**Table 3:** Radiographic results after 10 years follow-up

Radiographical parameter	
CH	73.8%
Bone condensation	100%
Cortical thinning	80.0%
Radiolucency	17.5%
Reactive lines	10.1%
Calcar rounding	86.2%
Calcar resorption	3.8%
Mean subsidence [mm] (± SD)	5.0 (± 3.1)

## Results

### Clinical results

**Fig. 3:**
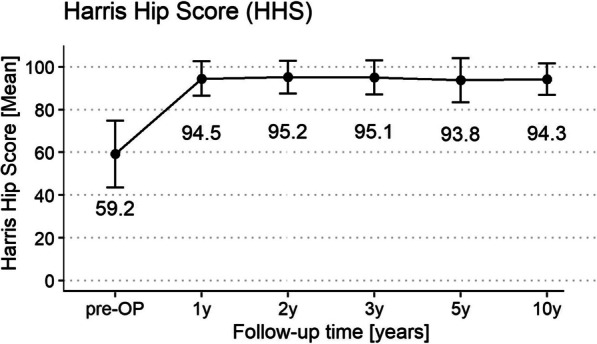
Harris Hip Score displayed as mean (± SD) over time course of 10 years

Clinical results are displayed in Table 2. There were no implant failures and the survival rate was 99% with 1 stem-related revision (1 aseptic loosening after 18 months which was treated by conversion to a straight uncemented CLS stem). 80 hips were evaluated at the final 10-year follow-up. Complications included 1 hip dislocation, which occurred after mobilization on the first postoperative day and was treated with closed reduction; 1 postoperative hematoma on the 11th day, which was resolved with conservative therapy; 1 anterior impingement, which was treated with an exchange of the acetabular cup; and 1 deep wound infection after 7 days, which was successfully treated with open debridement, irrigation, and antibiotics without the necessity of implant removal. Most patients experienced no (86%) or only slight (13%) thigh pain (Table 2). Similarly, most patients experienced no (70%) or at most moderate pain (27%) on the EQ5D-severity scale (Table 2). The HHS improved from 59.2 ± 15.6 to 94.3 ± 7.3 (Fig. 3) and the Oxford Hip Score improved from 22.2 ± 8.5 to 43.0 ± 5.5 after 10 years (Table 2).

### Radiographic results

10-year results are summarized in Table 3. The rate of CH after one year was 69%, increasing to 70% over five years and 74% after 10 years. Bone condensation increased from 79% after one year to 98% after 5 years and 100% after 10 years. Cortical thinning was 65% after one year, increasing to 73% after 5 years and 80% after 10 years. Radiolucency was 58% in the first year, diminishing to 37% after 5 years and 18% over 10 years. Reactive lines were seen in 44% of cases one year postoperatively, 17% after 5 years and 10% after 10 years. Calcar rounding increased from 70% after 1 year to 82% after 5 years and 86% after 10 years. Instead, calcar resorption decreased from 8% after 1 year to 4% both after 5 and 10 years. Mean Subsidence was 1.6 ± 1.6 mm after 1 year and increased gradually to 5.0 ± 3.1 mm after 10 years. Bone condensation (100%), cortical thinning (80%), and calcar rounding (86%) occurred frequently in the 10-year follow-up.

### Correlation between patient, implant, or surgical factors and radiographic outcome

None of the patient-, implant- or surgical parameters listed in Table 1 correlated with the radiographic outcome after 10 years (Table 3). Furthermore, there was no correlation between the radiographic parameters, e.g., cortical hypertrophy, and the clinical outcome after 10 years.

## Discussion

This is the first cohort-study reporting survival, clinical and radiographical results of Fitmore short hip stems after 10 years. HHS and Oxford Hip Score had improved constantly over time, showing a good long-term outcome with only one stem-related revision due to aseptic loosening in the study collective within 10 years.

In our study, the survival rate was 99% after 10 years with 1 stem-related revision out of 80 operated hips due to aseptic loosening. Similarly, Innmann et al. report a survival rate of 99.6% after 8.6 years for the Fitmore stem with revision due to aseptic loosening [12]. A long-term registry study compared survival rates of short versus conventional stems and showed comparable survival rates at long-term follow-up (> 90% at 15 years) with similar rates of stem aseptic loosening, intraoperative fractures, and periprosthetic fractures [13]. Furthermore, in the Dutch Arthroplasty Register, short stems like Fitmore and Optimys showed comparable revision rates after 10 years (3%) to standard-stem total hip replacements (2.3%) [14]. However, short stems other than Fitmore or Optimys had a higher revision rate of 4.5% [14].

The clinical findings were similar to those of other studies investigating long-term outcome of short hip stems. Capone et al. described a mean HHS of 90 (range 71–100) and no thigh pain after implantation of a Nanos short hip stem (Smith and Nephew, Marl, Germany) with a mean follow-up of 5.6 years (range 3–10 years) [15]. Another study compared an ultra-short with a cementless anatomic femoral stem of conventional length and reports no significant differences between the 2 groups in terms of the HHS (92 ± 6 vs. 91 ± 7 points, *P* = 0.173) at a mean follow-up of 16.5 years (range 15–18) in the ultra-short stem group and 17.5 years (range 17–20) in the conventional stem group [16]. Another study presented an 11-year follow-up of the anatomic coated CFP (Collum Femoris Preserving) Stem (Waldemar Link GmbH, Hamburg, Germany) with an improvement of HHS from 53 to 93 points, stem-related revisions due to aseptic loosening of 3.4% and a survival rate for the femoral component of 98.3% [17]. For the same implant Hutt et al. described an HHS improvement from a mean of 50 preoperatively to 91 (*p* < 0.001) postoperatively; no stem required revision and the survival rate was 100% for the stem at 10-year follow-up [18].

The design idea behind the short-stem prosthesis is to aim for a more even load transfer to the femur [3, 19]. Since a different load transfer results in bone remodeling, various radiographic alterations have been seen in our study. Cortical hypertrophy (74%), cortical thinning (80%) and subsidence (5.0 mm (± 3.1)), among others, were progressing over the course of the 10-year follow-up, whereas radiolucent lines (RLL) decreased. These findings are in line with the findings of other studies investigating radiographic alterations, even though they described a shorter follow-up period. A recent study [20] evaluated changes of radiographic findings up to three years after implantation of the Fitmore stem: CH was observed in 49 hips (21.1%); cortical thinning was observed in 63 hips (27.2%); and RLLs were observed in 34 hips (14.7%) one year postoperatively. Among 34 hips with RLLs, 70.6% did not progress or resolved on the three-year radiograph. Bone condensation was observed more frequently in younger patients [20]. Furthermore, Freitag et al. [21] reported subsidence until 2 years postoperative with settling of all stems afterward. However, in our study radiographic alterations did not affect clinical outcomes.

This study has certain limitations. The study had a relatively small sample size mainly due to patients lost to follow-up, refusal to participate, and death. On the other hand, the study’s strengths are a high data quality, a prospective study design, and the absence of conflict of interest. Long-term results of modern short hip stems are scarce in the current literature and this study is the first to report 10-year radiographic and clinical results of the cementless, metaphyseal anchored short Fitmore Hip Stem.

## Conclusion

10-year results of the Fitmore short hip stem showed good results regarding survival, and clinical and radiological data are comparable to standard stem designs in the long-term follow-up of 10 years. Various radiographic alterations like an increase in cortical hypertrophy and subsidence accompanied by a decrease in radiolucency were observed during 10-year follow-up of the Fitmore short hip stem. Patient-reported outcome measures and clinical outcome did not differ between patients with or without radiographic changes.

## Data Availability

The datasets used and/or analyzed during the current study are available from the corresponding author upon request.
